# Bis[μ-*N*′-(2-oxidobenzyl­idene)thio­phene-2-carbohydrazidato]bis­[dimethanol­nickel(II)]

**DOI:** 10.1107/S160053681102143X

**Published:** 2011-06-11

**Authors:** Jian-Hua Ma, Wen-Shi Wu, Xiao-Qing Zhang, Sheng-Jiao Tang, Xin-Yong Lin

**Affiliations:** a College of Materials Science and Engineering, Huaqiao University, Xiamen, Fujian 361021, People’s Republic of China

## Abstract

In the crystal structure of the centrosymmetric binuclear title complex, [Ni_2_(C_12_H_8_N_2_O_2_S)_2_(CH_3_OH)_4_], there are inter­molecular O—H⋯O, O—H⋯N and O—H⋯S hydrogen bonds. These help to stabilize the structure and link the mol­ecules, forming a three-dimensional network structure. The Ni^2+^ cation exists in a slightly distorted octahedral NiNO_5_ coordination environment. The thio­phene rings are disordered over two equivalent conformations with occupancies of 0.881 (3) and 0.119 (3).

## Related literature

For the structure of the related Cu complex, see: Lu *et al.* (2006 ▶). For the synthesis of the ligand, see: Wu *et al.* (2004 ▶).
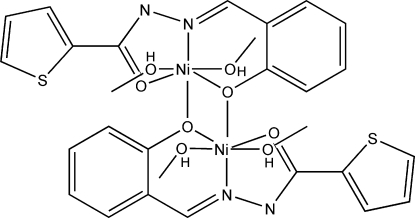


## Experimental

### 

#### Crystal data

[Ni_2_(C_12_H_8_N_2_O_2_S)_2_(CH_4_O)_4_]
*M* *_r_* = 734.12
Monoclinic, 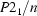

*a* = 13.7958 (14) Å
*b* = 7.8880 (8) Å
*c* = 14.4219 (16) Å
β = 104.672 (1)°
*V* = 1518.2 (3) Å^3^
*Z* = 2
Mo *K*α radiation
μ = 1.43 mm^−1^
*T* = 293 K
0.60 × 0.41 × 0.39 mm

#### Data collection

Bruker SMART CCD diffractometer
Absorption correction: multi-scan (*SADABS*; Sheldrick, 1996 ▶) *T*_min_ = 0.499, *T*_max_ = 0.572
11412 measured reflections
3484 independent reflections
3137 reflections with *I* > 2σ(*I*)
*R*_int_ = 0.029

#### Refinement

*R*[*F* ^2^ > 2σ(*F* ^2^)] = 0.037
*wR*(*F* ^2^) = 0.089
*S* = 1.11
3469 reflections
217 parameters
10 restraints
H-atom parameters constrained
Δρ_max_ = 0.36 e Å^−3^
Δρ_min_ = −0.34 e Å^−3^

### 

Data collection: *SMART* (Bruker, 1999 ▶); cell refinement: *SAINT* (Bruker, 1999 ▶); data reduction: *SAINT*; program(s) used to solve structure: *SHELXS97* (Sheldrick, 2008 ▶); program(s) used to refine structure: *SHELXL97* (Sheldrick, 2008 ▶); molecular graphics: *SHELXTL* (Sheldrick, 2008 ▶); software used to prepare material for publication: *SHELXTL*.

## Supplementary Material

Crystal structure: contains datablock(s) global, I. DOI: 10.1107/S160053681102143X/bv2181sup1.cif

Structure factors: contains datablock(s) I. DOI: 10.1107/S160053681102143X/bv2181Isup2.hkl

Additional supplementary materials:  crystallographic information; 3D view; checkCIF report

## Figures and Tables

**Table 1:** Selected bond lengths (Å)

Ni1—N2	1.9906 (18)
Ni1—O2	2.0165 (16)
Ni1—O1	2.0244 (16)
Ni1—O2^i^	2.0521 (15)
Ni1—O3	2.1425 (17)
Ni1—O4	2.1897 (16)

Symmetry code: (i) 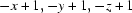.

**Table 2:** Hydrogen-bond geometry (Å, °)

*D*—H⋯*A*	*D*—H	H⋯*A*	*D*⋯*A*	*D*—H⋯*A*
O3—H16⋯O4^i^	0.82	2.13	2.920 (2)	163
O4—H15⋯N1^ii^	0.82	2.07	2.850 (2)	159
O4—H15⋯S1*A*^ii^	0.82	2.92	3.452 (3)	125

Symmetry codes: (i) 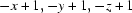; (ii) 
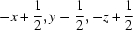
.

**Fig. 1.**
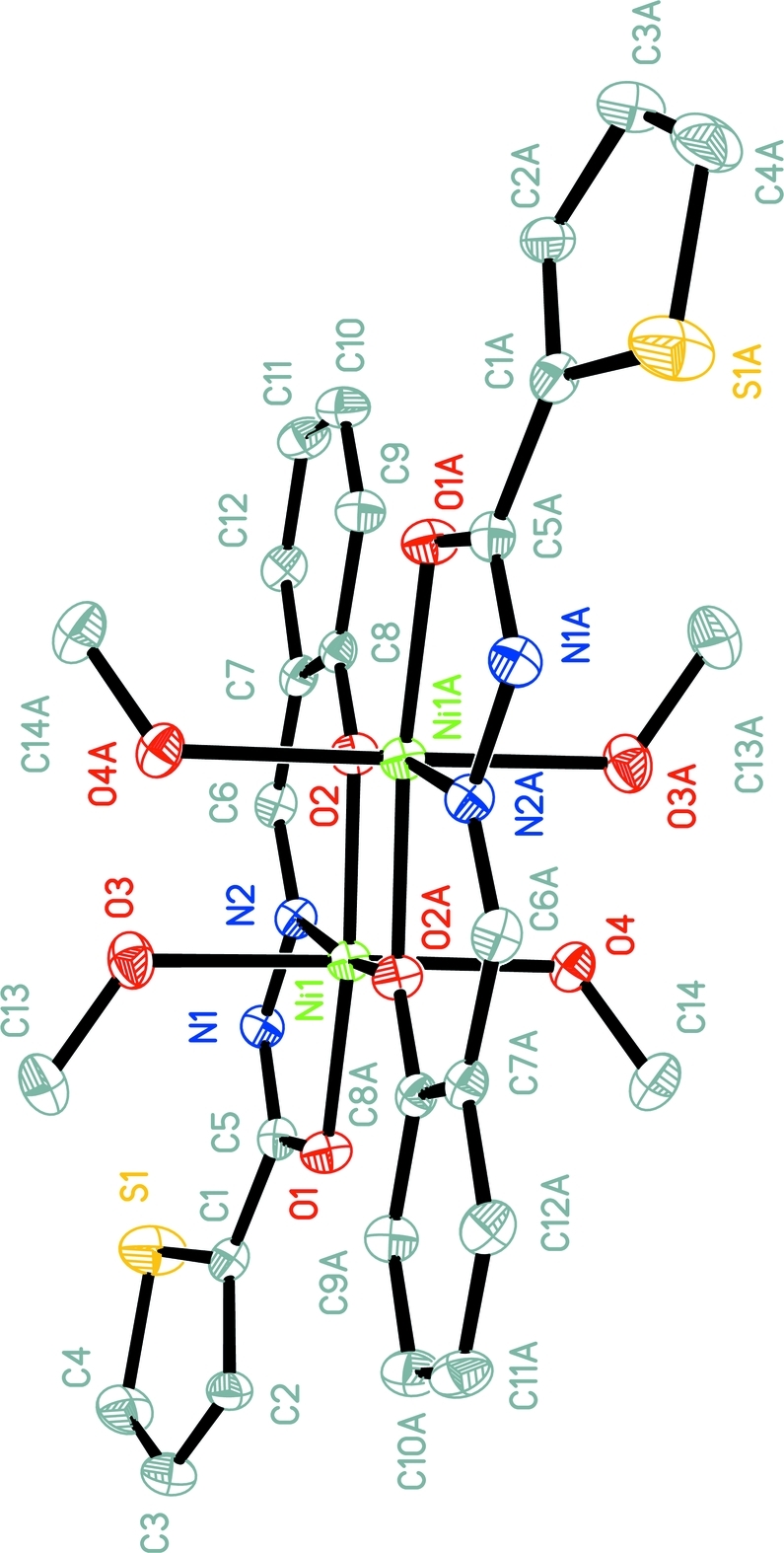
The molecular structure (at 30% probability) of the title compound.

**Fig. 2.**
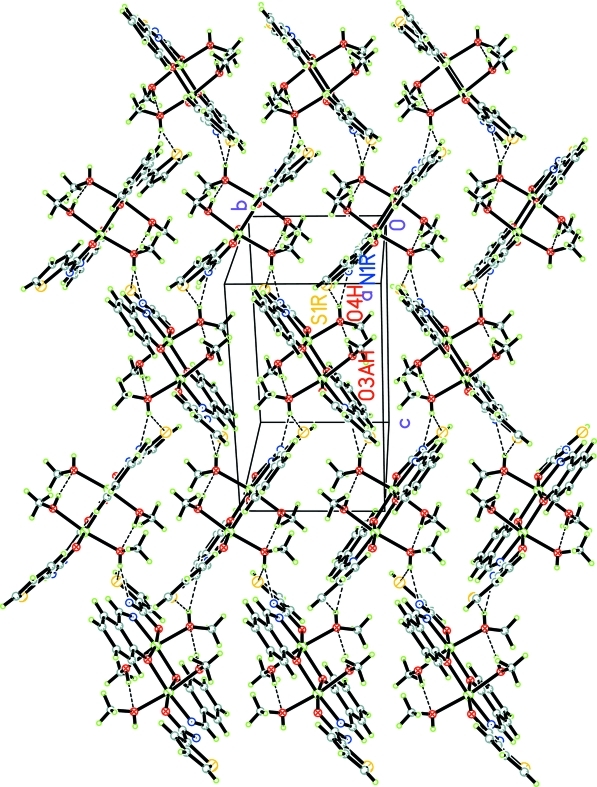
Packing diagram of the title complex, showing hydrogen bonds as dashed lines.

## supplementary crystallographic information

### Comment

The structure of [Cu(C_12_H_9_N_2_O_2_S)Cl].H_2_O (II), which is closely related to the title comlex has already been reported (Lu *et al.*, 2006). In this article, we report the crystal structure of the realted nickel complex, I.

In the title complex, [Ni_2_(C_24_H_16_N_4_O_4_S_2_)C_4_H_16_O_4_] (Fig.1 and Table 1), the Ni(II) cation is six-coordinated by an amide N, a phenoxide O and a carbonyl O atom derived from the first tridentate ligand, another phenoxide O atom derived from the second tridentate ligand and two other O atoms from two molecules of methanol. The five-and six-membered chelate rings are coplanar, the mean deviation of 0.0497 Å from the least-squares plane through both of them, slightly larger than that of the structure of II (0.037 (18)Å, Lu *et al.*). The thiophene rings are slightly twisted with respect to the above mentioned plane, making a larger dihedral angle of 15.4 (2)° than that of II (8.77 (9)°). The Ni-O and Ni-N distances [Ni—O1 (2.024 (2)Å), Ni—O2(2.016 (2)Å) and Ni—N2 (1.991 (2)Å)] are longer than the corresponding distances for II (Lu *et al.*, 2006). The O1—Ni1—N2 (79.53 (7)°) and O2—Ni1—N2 (91.24 (7)°) angles differ slightly from the corresponding angles found in II. The thiophene rings are disordered over two eqivalent conformations with ocuupancies of 0.881 (3) and 0.119 (3) as is commonly found for this moiety.

N—H···O, N—H···S and O—H···O intermolecular hydrogen bonds in the compound stabilize the structure and link the molecules in a three-dimensional network structure (shown in Fig.2) and detailed in Table 2.

### Experimental

The ligand was synthesized according to the method of Wu *et al.*,(2004). NiCl_2 _(1 mmol) and the ligand were separately dissolved in 20 ml methanol and the resulting solutions mixed slowly, stirred at room temperature for one hour, and then filtered. The filtrate was allowed to stand at room temperature for ten days, yielding deep-blue crystals of the title compound by slow evaporation.

### Refinement

The C-bound H atoms were included in the riding model approximation with C—H = 0.93 - 0.96 Å, all these H atoms included in the final refinement. The *U*_iso_ of each H atom = 1.2*U*_eq_(C) [1.5*U*_eq_(C) for CH_3_]. The methanol H atoms were refined isotropically.

### Crystal data

**Table:**

[Ni_2_(C_12_H_8_N_2_O_2_S)_2_(CH_4_O)_4_]	*F*(000) = 760
*M**_r_* = 734.12	*D*_x_ = 1.606 Mg m^−^^3^
Monoclinic, *P*2_1_/*n*	Mo *K*α radiation, λ = 0.71073 Å
Hall symbol: -P 2yn	Cell parameters from 1552 reflections
*a* = 13.7958 (14) Å	θ = 3.1–27.5°
*b* = 7.8880 (8) Å	µ = 1.43 mm^−^^1^
*c* = 14.4219 (16) Å	*T* = 293 K
β = 104.672 (1)°	Columnar, blue
*V* = 1518.2 (3) Å^3^	0.60 × 0.41 × 0.39 mm
*Z* = 2	

### Data collection

**Table:**

Bruker SMART CCD diffractometer	3484 independent reflections
Radiation source: fine-focus sealed tube	3137 reflections with *I* > 2σ(*I*)
graphite	*R*_int_ = 0.029
Detector resolution: 0 pixels mm^-1^	θ_max_ = 27.5°, θ_min_ = 3.1°
ω scans	*h* = −17→17
Absorption correction: multi-scan (*SADABS*; Sheldrick, 1996)	*k* = 0→10
*T*_min_ = 0.499, *T*_max_ = 0.572	*l* = 0→18
11412 measured reflections	

### Refinement

**Table:**

Refinement on *F*^2^	Primary atom site location: structure-invariant direct methods
Least-squares matrix: full	Secondary atom site location: difference Fourier map
*R*[*F*^2^ > 2σ(*F*^2^)] = 0.037	Hydrogen site location: inferred from neighbouring sites
*wR*(*F*^2^) = 0.089	H-atom parameters constrained
*S* = 1.11	*w* = 1/[σ^2^(*F*_o_^2^) + (0.0375*P*)^2^ + 0.8784*P*] where *P* = (*F*_o_^2^ + 2*F*_c_^2^)/3
3469 reflections	(Δ/σ)_max_ < 0.001
217 parameters	Δρ_max_ = 0.36 e Å^−^^3^
10 restraints	Δρ_min_ = −0.34 e Å^−^^3^

### Special details

**Table:**

Experimental. 2011-03-01 # Formatted by IUCr publCIF system
Geometry. All esds (except the esd in the dihedral angle between two l.s. planes) are estimated using the full covariance matrix. The cell esds are taken into account individually in the estimation of esds in distances, angles and torsion angles; correlations between esds in cell parameters are only used when they are defined by crystal symmetry. An approximate (isotropic) treatment of cell esds is used for estimating esds involving l.s. planes.
Refinement. Refinement of F^2^ against ALL reflections. The weighted R-factor wR and goodness of fit S are based on F^2^, conventional R-factors R are based on F, with F set to zero for negative F^2^. The threshold expression of F^2^ > 2sigma(F^2^) is used only for calculating R-factors(gt) etc. and is not relevant to the choice of reflections for refinement. R-factors based on F^2^ are statistically about twice as large as those based on F, and R- factors based on ALL data will be even larger.

### Fractional atomic coordinates and isotropic or equivalent isotropic displacement parameters (Å^2^)

**Table:**

	*x*	*y*	*z*	*U*_iso_*/*U*_eq_	Occ. (<1)
Ni1	0.39217 (2)	0.56046 (3)	0.449805 (19)	0.02409 (10)	
S1A	0.03964 (10)	0.9026 (2)	0.32607 (15)	0.0563 (5)	0.881 (3)
C1A	0.1016 (3)	0.7392 (5)	0.3958 (3)	0.0273 (7)	0.881 (3)
C2A	0.0491 (6)	0.6804 (10)	0.4605 (6)	0.0408 (11)	0.881 (3)
H2AA	0.0709	0.5952	0.5056	0.049*	0.881 (3)
C3A	−0.0430 (3)	0.7708 (5)	0.4469 (4)	0.0436 (9)	0.881 (3)
H3AA	−0.0897	0.7471	0.4816	0.052*	0.881 (3)
C4A	−0.0570 (3)	0.8928 (6)	0.3797 (3)	0.0495 (10)	0.881 (3)
H4AA	−0.1127	0.9635	0.3643	0.059*	0.881 (3)
S1B	0.0546 (13)	0.687 (2)	0.4652 (14)	0.0563 (5)	0.119 (3)
C1B	0.104 (3)	0.767 (5)	0.379 (3)	0.0273 (7)	0.119 (3)
C2B	0.053 (3)	0.914 (6)	0.338 (4)	0.0408 (11)	0.119 (3)
H2BA	0.0773	1.0035	0.3088	0.049*	0.119 (3)
C3B	−0.046 (2)	0.896 (5)	0.352 (2)	0.0436 (9)	0.119 (3)
H3BA	−0.1026	0.9367	0.3086	0.052*	0.119 (3)
C4B	−0.049 (3)	0.814 (6)	0.433 (3)	0.0495 (10)	0.119 (3)
H4BA	−0.0992	0.8237	0.4652	0.059*	0.119 (3)
O1	0.24595 (12)	0.5752 (2)	0.45082 (12)	0.0313 (4)	
O2	0.53297 (11)	0.57985 (18)	0.43430 (11)	0.0263 (3)	
O3	0.42607 (13)	0.7737 (2)	0.54454 (13)	0.0401 (4)	
H16	0.4807	0.7471	0.5794	0.060*	
O4	0.36612 (12)	0.3220 (2)	0.36958 (12)	0.0322 (4)	
H15	0.3433	0.3266	0.3113	0.048*	
N1	0.24055 (14)	0.7622 (2)	0.32434 (14)	0.0288 (4)	
N2	0.34061 (13)	0.7130 (2)	0.33827 (13)	0.0259 (4)	
C5	0.20199 (16)	0.6862 (3)	0.38915 (16)	0.0262 (4)	
C6	0.39124 (17)	0.7867 (3)	0.28636 (16)	0.0285 (5)	
H6A	0.3575	0.8637	0.2406	0.034*	
C7	0.49684 (16)	0.7591 (3)	0.29352 (16)	0.0260 (4)	
C8	0.56252 (16)	0.6577 (3)	0.36440 (15)	0.0244 (4)	
C9	0.66258 (17)	0.6423 (3)	0.35929 (17)	0.0317 (5)	
H9A	0.7066	0.5765	0.4047	0.038*	
C10	0.69723 (19)	0.7222 (3)	0.28877 (19)	0.0374 (6)	
H10A	0.7638	0.7088	0.2873	0.045*	
C11	0.63412 (19)	0.8220 (3)	0.22015 (18)	0.0375 (6)	
H11A	0.6578	0.8766	0.1731	0.045*	
C12	0.53578 (18)	0.8385 (3)	0.22323 (18)	0.0330 (5)	
H12A	0.4932	0.9049	0.1770	0.040*	
C13	0.3631 (3)	0.8355 (4)	0.6013 (3)	0.0574 (8)	
H13A	0.3956	0.9281	0.6402	0.086*	
H13B	0.3503	0.7462	0.6419	0.086*	
H13C	0.3009	0.8738	0.5602	0.086*	
C14	0.3199 (2)	0.1845 (3)	0.4077 (2)	0.0448 (6)	
H14A	0.2951	0.1025	0.3582	0.067*	
H14B	0.2654	0.2271	0.4311	0.067*	
H14C	0.3685	0.1319	0.4593	0.067*	

### Atomic displacement parameters (Å^2^)

**Table:**

	*U*^11^	*U*^22^	*U*^33^	*U*^12^	*U*^13^	*U*^23^
Ni1	0.02149 (15)	0.02730 (16)	0.02132 (16)	0.00196 (10)	0.00142 (11)	0.00409 (11)
S1A	0.0438 (7)	0.0593 (7)	0.0688 (10)	0.0245 (6)	0.0200 (7)	0.0337 (8)
C1A	0.0267 (11)	0.023 (2)	0.028 (2)	0.0005 (12)	0.0005 (12)	0.0056 (11)
C2A	0.036 (2)	0.047 (2)	0.043 (2)	−0.0097 (15)	0.0153 (15)	−0.0102 (16)
C3A	0.0327 (16)	0.051 (3)	0.050 (2)	0.0043 (15)	0.0156 (16)	0.0032 (17)
C4A	0.0349 (18)	0.054 (2)	0.062 (3)	0.0181 (15)	0.0165 (17)	0.009 (2)
S1B	0.0438 (7)	0.0593 (7)	0.0688 (10)	0.0245 (6)	0.0200 (7)	0.0337 (8)
C1B	0.0267 (11)	0.023 (2)	0.028 (2)	0.0005 (12)	0.0005 (12)	0.0056 (11)
C2B	0.036 (2)	0.047 (2)	0.043 (2)	−0.0097 (15)	0.0153 (15)	−0.0102 (16)
C3B	0.0327 (16)	0.051 (3)	0.050 (2)	0.0043 (15)	0.0156 (16)	0.0032 (17)
C4B	0.0349 (18)	0.054 (2)	0.062 (3)	0.0181 (15)	0.0165 (17)	0.009 (2)
O1	0.0240 (8)	0.0361 (9)	0.0320 (9)	0.0034 (6)	0.0040 (7)	0.0105 (7)
O2	0.0248 (8)	0.0310 (8)	0.0210 (8)	0.0017 (6)	0.0018 (6)	0.0074 (6)
O3	0.0373 (9)	0.0399 (10)	0.0386 (10)	0.0025 (7)	0.0015 (8)	−0.0098 (8)
O4	0.0360 (9)	0.0334 (8)	0.0236 (8)	−0.0058 (7)	0.0008 (7)	−0.0022 (7)
N1	0.0247 (9)	0.0312 (10)	0.0276 (10)	0.0051 (7)	0.0012 (8)	0.0040 (8)
N2	0.0222 (9)	0.0284 (9)	0.0239 (10)	0.0017 (7)	0.0000 (7)	0.0028 (7)
C5	0.0247 (10)	0.0264 (10)	0.0244 (11)	0.0013 (8)	0.0003 (9)	−0.0003 (8)
C6	0.0308 (11)	0.0272 (11)	0.0238 (11)	0.0025 (9)	−0.0001 (9)	0.0051 (9)
C7	0.0301 (11)	0.0241 (10)	0.0220 (11)	−0.0029 (8)	0.0034 (9)	0.0003 (8)
C8	0.0275 (11)	0.0249 (10)	0.0195 (10)	−0.0028 (8)	0.0035 (8)	−0.0023 (8)
C9	0.0274 (11)	0.0382 (12)	0.0280 (12)	0.0012 (9)	0.0045 (9)	0.0032 (10)
C10	0.0277 (12)	0.0489 (14)	0.0360 (14)	−0.0027 (10)	0.0088 (10)	0.0009 (11)
C11	0.0397 (14)	0.0458 (14)	0.0292 (13)	−0.0085 (11)	0.0127 (11)	0.0070 (11)
C12	0.0367 (13)	0.0327 (12)	0.0273 (12)	−0.0011 (10)	0.0042 (10)	0.0070 (9)
C13	0.065 (2)	0.0542 (18)	0.059 (2)	−0.0006 (15)	0.0263 (17)	−0.0225 (15)
C14	0.0552 (17)	0.0366 (13)	0.0403 (15)	−0.0131 (12)	0.0080 (13)	0.0013 (11)

### Geometric parameters (Å, °)

**Table:**

Ni1—N2	1.9906 (18)	O2—Ni1^i^	2.0521 (15)
Ni1—O2	2.0165 (16)	O3—C13	1.422 (3)
Ni1—O1	2.0244 (16)	O3—H16	0.8200
Ni1—O2^i^	2.0521 (15)	O4—C14	1.436 (3)
Ni1—O3	2.1425 (17)	O4—H15	0.8206
Ni1—O4	2.1897 (16)	N1—C5	1.330 (3)
S1A—C4A	1.704 (4)	N1—N2	1.399 (3)
S1A—C1A	1.722 (3)	N2—C6	1.285 (3)
C1A—C2A	1.397 (8)	C6—C7	1.451 (3)
C1A—C5	1.474 (4)	C6—H6A	0.9300
C2A—C3A	1.427 (8)	C7—C12	1.409 (3)
C2A—H2AA	0.9300	C7—C8	1.427 (3)
C3A—C4A	1.345 (5)	C8—C9	1.406 (3)
C3A—H3AA	0.9300	C9—C10	1.381 (3)
C4A—H4AA	0.9300	C9—H9A	0.9300
S1B—C1B	1.680 (18)	C10—C11	1.386 (4)
S1B—C4B	1.710 (18)	C10—H10A	0.9300
C1B—C2B	1.41 (2)	C11—C12	1.375 (4)
C1B—C5	1.47 (3)	C11—H11A	0.9300
C2B—C3B	1.43 (2)	C12—H12A	0.9300
C2B—H2BA	0.9300	C13—H13A	0.9600
C3B—C4B	1.344 (19)	C13—H13B	0.9600
C3B—H3BA	0.9300	C13—H13C	0.9600
C4B—H4BA	0.9300	C14—H14A	0.9600
O1—C5	1.285 (3)	C14—H14B	0.9600
O2—C8	1.330 (3)	C14—H14C	0.9600
			
N2—Ni1—O2	91.24 (7)	Ni1—O3—H16	102.0
N2—Ni1—O1	79.53 (7)	C14—O4—Ni1	118.42 (15)
O2—Ni1—O1	170.42 (6)	C14—O4—H15	109.5
N2—Ni1—O2^i^	170.91 (7)	Ni1—O4—H15	118.2
O2—Ni1—O2^i^	80.27 (7)	C5—N1—N2	109.27 (17)
O1—Ni1—O2^i^	108.78 (6)	C6—N2—N1	116.97 (18)
N2—Ni1—O3	90.93 (8)	C6—N2—Ni1	127.48 (15)
O2—Ni1—O3	87.16 (7)	N1—N2—Ni1	114.92 (14)
O1—Ni1—O3	90.41 (7)	O1—C5—N1	126.2 (2)
O2^i^—Ni1—O3	85.43 (7)	O1—C5—C1B	128.6 (10)
N2—Ni1—O4	96.61 (7)	N1—C5—C1B	105.0 (9)
O2—Ni1—O4	92.32 (6)	O1—C5—C1A	115.8 (2)
O1—Ni1—O4	91.30 (6)	N1—C5—C1A	117.9 (2)
O2^i^—Ni1—O4	87.05 (6)	N2—C6—C7	125.1 (2)
O3—Ni1—O4	172.44 (7)	N2—C6—H6A	117.4
C4A—S1A—C1A	91.74 (17)	C7—C6—H6A	117.4
C2A—C1A—C5	127.2 (4)	C12—C7—C8	118.4 (2)
C2A—C1A—S1A	112.0 (4)	C12—C7—C6	116.2 (2)
C5—C1A—S1A	120.6 (2)	C8—C7—C6	125.4 (2)
C1A—C2A—C3A	109.6 (6)	O2—C8—C9	119.57 (19)
C1A—C2A—H2AA	125.2	O2—C8—C7	122.76 (19)
C3A—C2A—H2AA	125.2	C9—C8—C7	117.7 (2)
C4A—C3A—C2A	114.6 (5)	C10—C9—C8	121.8 (2)
C4A—C3A—H3AA	122.7	C10—C9—H9A	119.1
C2A—C3A—H3AA	122.7	C8—C9—H9A	119.1
C3A—C4A—S1A	112.0 (3)	C9—C10—C11	120.9 (2)
C3A—C4A—H4AA	124.0	C9—C10—H10A	119.6
S1A—C4A—H4AA	124.0	C11—C10—H10A	119.6
C1B—S1B—C4B	92.8 (12)	C12—C11—C10	118.5 (2)
C2B—C1B—C5	139 (2)	C12—C11—H11A	120.7
C2B—C1B—S1B	111.3 (17)	C10—C11—H11A	120.7
C5—C1B—S1B	107.6 (17)	C11—C12—C7	122.7 (2)
C1B—C2B—C3B	105 (2)	C11—C12—H12A	118.6
C1B—C2B—H2BA	127.7	C7—C12—H12A	118.6
C3B—C2B—H2BA	127.7	O3—C13—H13A	109.5
C4B—C3B—C2B	114 (2)	O3—C13—H13B	109.5
C4B—C3B—H3BA	122.8	H13A—C13—H13B	109.5
C2B—C3B—H3BA	122.8	O3—C13—H13C	109.5
C3B—C4B—S1B	107.8 (18)	H13A—C13—H13C	109.5
C3B—C4B—H4BA	126.1	H13B—C13—H13C	109.5
S1B—C4B—H4BA	126.1	O4—C14—H14A	109.5
C5—O1—Ni1	109.30 (14)	O4—C14—H14B	109.5
C8—O2—Ni1	127.45 (13)	H14A—C14—H14B	109.5
C8—O2—Ni1^i^	132.76 (14)	O4—C14—H14C	109.5
Ni1—O2—Ni1^i^	99.73 (7)	H14A—C14—H14C	109.5
C13—O3—Ni1	124.72 (17)	H14B—C14—H14C	109.5
C13—O3—H16	109.6		
			
C4A—S1A—C1A—C2A	−1.0 (5)	O1—Ni1—N2—N1	5.62 (14)
C4A—S1A—C1A—C5	−176.0 (4)	O3—Ni1—N2—N1	−84.62 (15)
C5—C1A—C2A—C3A	176.7 (5)	O4—Ni1—N2—N1	95.73 (15)
S1A—C1A—C2A—C3A	2.0 (7)	Ni1—O1—C5—N1	9.3 (3)
C1A—C2A—C3A—C4A	−2.3 (7)	Ni1—O1—C5—C1B	−165 (3)
C2A—C3A—C4A—S1A	1.6 (6)	Ni1—O1—C5—C1A	−167.0 (3)
C1A—S1A—C4A—C3A	−0.4 (4)	N2—N1—C5—O1	−4.8 (3)
C4B—S1B—C1B—C2B	12 (5)	N2—N1—C5—C1B	171 (2)
C4B—S1B—C1B—C5	178 (3)	N2—N1—C5—C1A	171.5 (3)
C5—C1B—C2B—C3B	175 (5)	C2B—C1B—C5—O1	159 (5)
S1B—C1B—C2B—C3B	−26 (5)	S1B—C1B—C5—O1	0(4)
C1B—C2B—C3B—C4B	33 (6)	C2B—C1B—C5—N1	−17 (7)
C2B—C3B—C4B—S1B	−25 (5)	S1B—C1B—C5—N1	−176 (2)
C1B—S1B—C4B—C3B	7(4)	C2B—C1B—C5—C1A	166 (16)
N2—Ni1—O1—C5	−7.40 (15)	S1B—C1B—C5—C1A	7(8)
O2^i^—Ni1—O1—C5	168.79 (14)	C2A—C1A—C5—O1	−1.0 (7)
O3—Ni1—O1—C5	83.47 (15)	S1A—C1A—C5—O1	173.2 (3)
O4—Ni1—O1—C5	−103.89 (15)	C2A—C1A—C5—N1	−177.7 (5)
N2—Ni1—O2—C8	−5.77 (17)	S1A—C1A—C5—N1	−3.4 (5)
O2^i^—Ni1—O2—C8	177.5 (2)	C2A—C1A—C5—C1B	−174 (11)
O3—Ni1—O2—C8	−96.64 (17)	S1A—C1A—C5—C1B	0(10)
O4—Ni1—O2—C8	90.90 (17)	N1—N2—C6—C7	176.82 (19)
N2—Ni1—O2—Ni1^i^	176.73 (7)	Ni1—N2—C6—C7	6.4 (3)
O2^i^—Ni1—O2—Ni1^i^	0.0	N2—C6—C7—C12	173.4 (2)
O3—Ni1—O2—Ni1^i^	85.85 (7)	N2—C6—C7—C8	−5.7 (4)
O4—Ni1—O2—Ni1^i^	−86.60 (7)	Ni1—O2—C8—C9	−172.88 (15)
N2—Ni1—O3—C13	97.2 (2)	Ni1^i^—O2—C8—C9	3.8 (3)
O2—Ni1—O3—C13	−171.6 (2)	Ni1—O2—C8—C7	7.7 (3)
O1—Ni1—O3—C13	17.7 (2)	Ni1^i^—O2—C8—C7	−175.67 (15)
O2^i^—Ni1—O3—C13	−91.1 (2)	C12—C7—C8—O2	179.0 (2)
N2—Ni1—O4—C14	−134.19 (17)	C6—C7—C8—O2	−1.8 (3)
O2—Ni1—O4—C14	134.29 (17)	C12—C7—C8—C9	−0.4 (3)
O1—Ni1—O4—C14	−54.58 (17)	C6—C7—C8—C9	178.7 (2)
O2^i^—Ni1—O4—C14	54.16 (17)	O2—C8—C9—C10	−179.3 (2)
C5—N1—N2—C6	−174.3 (2)	C7—C8—C9—C10	0.2 (3)
C5—N1—N2—Ni1	−2.7 (2)	C8—C9—C10—C11	0.3 (4)
O2—Ni1—N2—C6	−1.2 (2)	C9—C10—C11—C12	−0.6 (4)
O1—Ni1—N2—C6	176.2 (2)	C10—C11—C12—C7	0.4 (4)
O3—Ni1—N2—C6	85.9 (2)	C8—C7—C12—C11	0.1 (4)
O4—Ni1—N2—C6	−93.7 (2)	C6—C7—C12—C11	−179.1 (2)
O2—Ni1—N2—N1	−171.80 (14)		

Symmetry codes: (i) −*x*+1, −*y*+1, −*z*+1.

### Hydrogen-bond geometry (Å, °)

**Table:**

*D*—H···*A*	*D*—H	H···*A*	*D*···*A*	*D*—H···*A*
O3—H16···O4^i^	0.82	2.13	2.920 (2)	163.
O4—H15···N1^ii^	0.82	2.07	2.850 (2)	159.
O4—H15···S1A^ii^	0.82	2.92	3.452 (3)	125.

Symmetry codes: (i) −*x*+1, −*y*+1, −*z*+1; (ii) −*x*+1/2, *y*−1/2, −*z*+1/2.
